# Analysis of the enzyme network involved in cattle milk production using graph theory

**Published:** 2015-06

**Authors:** Sholeh Ghorbani, Mojtaba Tahmoorespur, Ali Masoudi Nejad, Mohammad Nasiri, Yazdan Asgari

**Affiliations:** 1 Ferdowsi University of Mashhad, Mashhad, Iran; 2 Institute of Biochemistry and Biophysics, University of Tehran, Tehran, Iran

**Keywords:** Cattle, Milk Production, Enzyme Network, Graph Theory, Metabolism

## Abstract

Understanding cattle metabolism and its relationship with milk products is important in bovine breeding. A systemic view could lead to consequences that will result in a better understanding of existing concepts. Topological indices and quantitative characterizations mostly result from the application of graph theory on biological data. In the present work, the enzyme network involved in cattle milk production was reconstructed and analyzed based on available bovine genome information using several public datasets (NCBI, Uniprot, KEGG, and Brenda). The reconstructed network consisted of 3605 reactions named by KEGG compound numbers and 646 enzymes that catalyzed the corresponding reactions. The characteristics of the directed and undirected network were analyzed using Graph Theory. The mean path length was calculated to be4.39 and 5.41 for directed and undirected networks, respectively. The top 11 hub enzymes whose abnormality could harm bovine health and reduce milk production were determined. Therefore, the aim of constructing the enzyme centric network was twofold; first to find out whether such network followed the same properties of other biological networks, and second, to find the key enzymes. The results of the present study can improve our understanding of milk production in cattle. Also, analysis of the enzyme network can help improve the modeling and simulation of biological systems and help design desired phenotypes to increase milk production quality or quantity.

## INTRODUCTION

Graph theory is the study of graphs that can be used to model relationships in different types of systems (such as biological and social information and so on). In the recent decades, great achievements have been made in the developing theory of biological networks [1]. One of the outreaching goals of systems' biology is the study of complex biological networks (gene, protein, metabolic networks, etc.) [2-3].

Many practical problems in biological systems could be represented by graphs. A graph G (V; E) is a set V of vertices and a set E of edges. Graphs could be defined as undirected or directed based on their edges. Metabolic networks, as a group of biological networks, could also be represented by graphs. Metabolism contains the sum of all biochemical reactions catalyzed by enzymes in a cell. Chemical reactions of metabolism are organized into metabolic pathways [4]. Metabolic networks are among the most studied biochemical networks [5]. Large scale metabolic reconstruction provides a highly mathematical, structured platform that enables biological science to proceed in fundamental new ways [6]. The study of methods for developing metabolic reconstructions has been reviewed in recent years [7]. At the moment, metabolic databases such as KEGG are available to reconstruct an organism specific metabolic network from genome information using several methods [8]. Based on the graph theory and depending on a metabolic network nodes types, metabolic networks could be classified as: metabolite networks (metabolites as nodes), enzyme networks (enzymes as nodes), and bipartite networks (both metabolites and enzymes as nodes) [9-10].

Despite the rapid development of systems' biology, studies on mammals are still rare, especially those focusing on large-scale metabolic networks of livestock [6]. Large- scale metabolic network studies can help develop animal sciences. Cattle milk, which itself considers as part of a metabolic network, is an agro-economical product and an essential human food; thus, an improvement in dairy cattle milk production is important.

Traditionally, genes associated with milk production traits are individually studied and elites are selected based on their genotypes in these loci [11-13]. This method is both costly and time consuming. Comparatively, the integration of knowledge at the metabolic level in a large-scale network requires less labor and time. Hence, this process is pivotal for the in-depth understanding and improvement of milk yield. Presently, the reconstruction of a large-scale metabolic network of dairy cattle has become possible, and the whole genome sequence for cattle has been published [14]. Human tissue specific network reconstruction encouraged us to focus on the reconstruction of an enzyme network involved in milk production in cattle (tissue- specific network) [15].

Using Graph Theory, in the present work the enzyme network involved in milk production in cattle was analyzed using the available genome annotation. Cattle mammary gland tissue has multiple metabolic potentials for large-scale synthesis of milk proteins, carbohydrates, and lipids, including nutrient triacylglycerols [5]. Milk production can be studied by reconstructing the metabolic network in mammary gland tissue using system biology methods. The present study can help enhance our understanding of cattle milk production.

## MATERIALS AND METHODS

A total of 6,875 expressed mammary gland tissue-specific genes involved in cattle milk production were downloaded from the UniGene database by ftp service. Lemay et al. reported 6,469 genes [16]. Each gene was queried in the Uniprot database to verify whether it was an enzyme. At this step, 791 enzyme-encoding genes were detected. Catalytic functions of each enzyme are generally described through the EC numbers assigned to catalyzed reactions [17]. The corresponding reaction information was queried in KEGG and Brenda databases and all reactions with substrates and products were selected [18]. Finally, 791 bovine mammary gland genes were found to account for the 2050 reaction formulas in 646 enzymes. Based on the data, we wrote a Programe in C# in order to extract the information to a desired format convertible to the Systems Biology Markup Language [SBML] format [19].

There were ten compartments [cytoplasm, extracellular space, mitochondria, Golgi apparatus, endoplasmic reticulum, lysosome, peroxisome, Cytosol, Vacuol and nucleus], accounting for 3065 reactions and 5837 metabolites. The SCAN-toolbox package [10] was used to construct directed and undirected reaction-centric networks based on an SBML file. The reaction-centric network must be built on the SBML file because it contains a bipartite graph. This toolbox contains a set of MATLAB scripts that take an SBML file as input. An important point in reaction-centric networks is that currency metabolites should be removed [20]. In our study, these metabolites were: ADP, ATP, CO2, O2, H2O, H2O2, H+, NAD, NADH, NADP, NADPH, and NH4. The global network was reconstructed using the publicly available Cytoscape software [21]. The topological attributes of the network and parameters of nodes, including degree, mean path length, network diameter, etc. were analyzed using a number of Cytoscapeplugins such as Network Analysis and cytoHubba [2].

## RESULTS AND DISCUSSION

Graph theory includes methods that have been proven beneficial for network topological analysis [22-23]. Real networks display a scale-free property, and a significant difference has been found between random and scale-free networks. Topological characteristics of the constructed networks were analyzed using the Network Analysis plugin and parameters of each node were also calculated. General characteristics of the networks are shown in table 1. The network file is attached in Supplementary file 2 and 3, and it can be viewed using the Cytoscape software.

**Figure 1:**
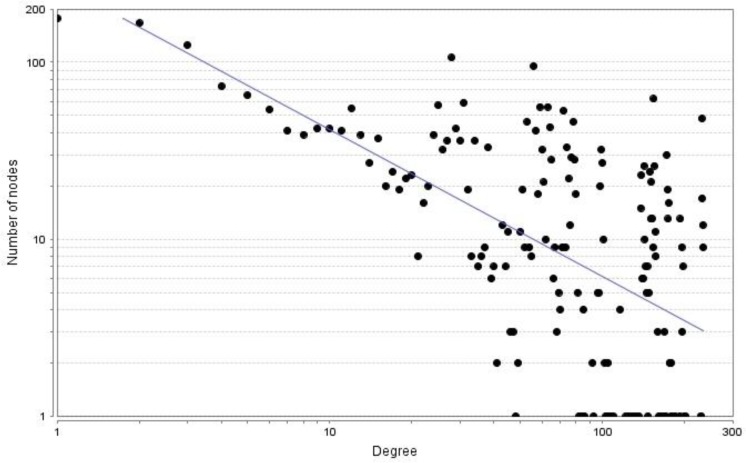
Degree distribution of the undirected network conforms to a power law, with the correlationcoefficient of r = 0.36 (P<0.0001). The value of γ is as small as 0.83. The degree of distribution displays a scale-free property of the network

**Figure 2:**
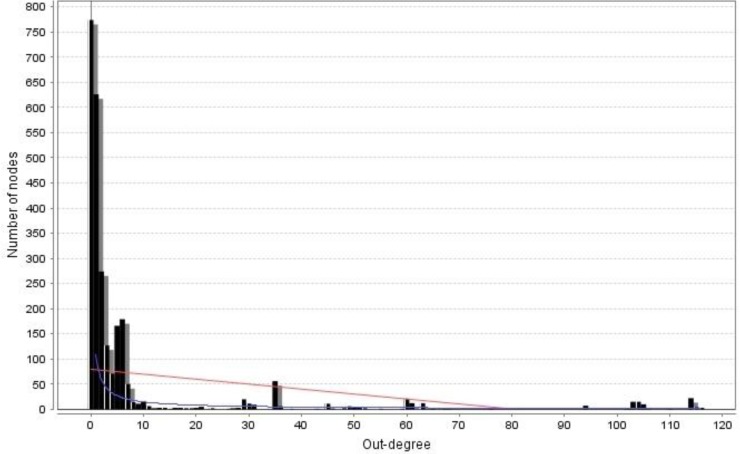
Degree distribution of the directed network conforms to a power law, with the correlation coefficient of r = 0.46 and 0.36 (P<0.0001). The value of γ is as small as 1.08 and 0.87(in-degree and out-degree respectively). The degree of distribution displays a scale-free property of the network

An enzyme centric network is constructed as the vertices of the graph are enzymes and an edge is considered if there is at least one common metabolite between two enzymes. [13]. The degree of node and degree distribution are considered as the most used topological characteristics of a network. The degree of node corresponds to the number of nodes neighboring a given node v, where neighbor means directly connected [24]. Determining the degree distribution allows for the discrimination of network classes [25]. The degree distribution of a real network follows the power-law distribution [26]:

*P*[*k*] ~ *k*-γ,

where the superscript γ is the power-law coefficient that determines many properties of

the system. The smaller the value of γ, the more important the role of the "hub" nodes in the network [25]. The degree distribution of the constructed undirected network is shown in Fig. 1, where the γ is 0.83 and correlation coefficient *r *is 0.78 [P<0.0001]. The degree distribution of the constructed directed network is shown in Fig. 2, where γ is 1.08 and 0.87 for in-degree and out-degree, respectively, and the correlation coefficients *(r) *are 0.97 and 0.95 for in-degree and out-degree, respectively [P<0.0001]. Generally speaking, the irregular properties of scale-free networks are valid only for the exponent of a power-law [γ] <3. In the present work, the degree distribution graphs in Fig. 1 and 2 clearly indicate the scale free nature and power law behavior of the cattle enzyme network [27].

**Table 1:** General characteristics of the constructed networks

**Parameters**	**Directed Values**	**Undirected Values**
Nodes	2614	3198
Edges	21891	89639
Characterististic Path Length	4.39	5.41
Network diameter	18	16
Clustering Coefficient	0.04	0.83

These parameters are also important characteristics because they offer a measure of a network’s overall navigability and show how high and low are better defined when compared to the total number of nodes in the graph. For the whole network, the diameter is the largest distance between two nodes which shows the development of the network in time, while the mean path length is the average length of the shortest path between any pair of nodes [25]. Thus, a biological network with a large size and low parameters may suggest that the proteins within the network had a functional co- evolution [1].

**Figure 3:**
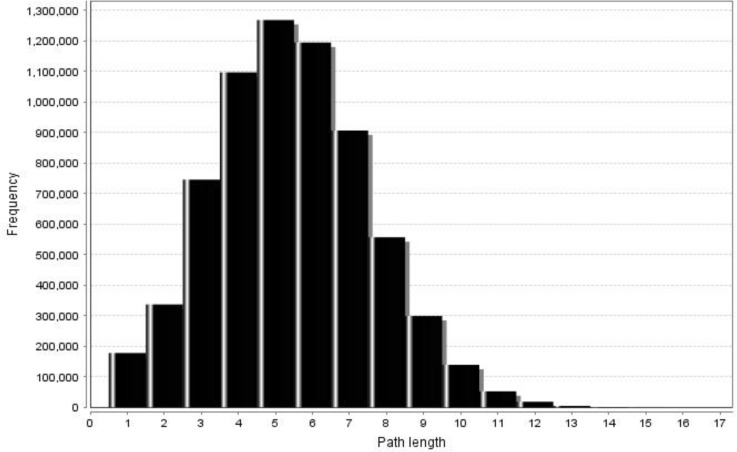
Shortest path length distribution of the directed network conforms to the normal distribution. For the entire network, the value of the mean path length is equal to 4.39

**Figure 4:**
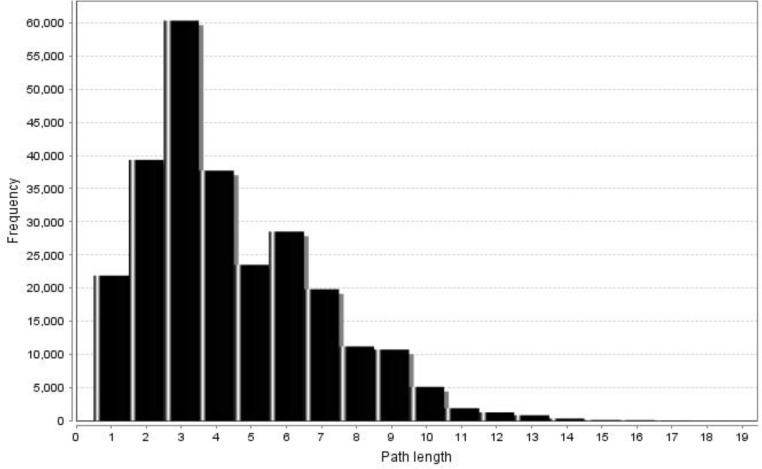
Shortest path length distribution of the undirected network conforms to the normal distribution. For the entire network, the value of the mean path length is equal to 5.41

Figures 3 and 4 (undirected and directed networks) show that in the present network, the shortest path length distributions conformed to a normal distribution (the path length characteristic being equal to 5.41 and 4.39 for undirected and directed networks, respectively). In addition, network diameter values were 16 and 18 for undirected and directed networks, which were much larger than that of a random network [28]. Previous studies revealed that many metabolic networks had a similar mean path length of approximately 3.2, which is almost equal to the values obtained by the present study, suggesting that metabolic networks are small-world networks [23]. In spite of this, Ma and Zeng reconstructed the metabolic networks of 80 organisms and maintained that such finding is not biochemically significant. They also showed that eukaryotes and archaea had longer average path lengths than bacteria [20]. In our study, these two important network properties (mean path length and network diameter) were approximately equal to the values of bacteria (7.23 and 20.6), and much larger or smaller than eukaryotes (9.57 and 33.1) and archaea (8.50 and 23.4). This indicates that although the primary structure of metabolic networks is similar for all organisms, they have a different evolutionary history [20].

We analyzed the directed network with the cytoHubba plug-in [2] and identified 11 hub enzymes. Table 2 shows the list of the top ten high degree enzymes. Corresponding genes as well as the involved pathways and reactions are shown in Table 3. The associated pathways seem to be useful and essential for cattle breeding research. Since hubs in scale-free networks play a significant role in maintaining topological robustness [29], we attempted to identify hub nodes that dominated the network structure (Table 2). Following the Barabasi-Albert algorithm of the preferential growth model, hub enzymes (with high degrees nodes) are likely to be ancient enzymes [30]. Therefore, high degree enzymes that belong to the primitive class of the enzyme network should be highly conserved [30-31]. These hub enzymes play an important biological role in the mammary glands' milk production and, therefore need to be studied further [13-32].

**Table 2:** First 11 hub enzymes of the metabolic networks ranked by the degree of the nodes

**Hub Enzyme**	**Degree**
Superoxide dismutase 2, mitochondrial	174
Cytochrome P450 2D14	172
Gamma-glutamyl carboxylase	151
Alpha-aminoadipic semialdehyde dehydrogenase	136
Trans-2-enoyl-CoA reductase, mitochondrial	135
Dihydropteridine reductase	133
Fatty acid synthase	129
Thioredoxin reductase 2, mitochondrial	129
Thioredoxin reductase 1, cytoplasmic (TR)	129
Prostaglandin reductase 1	127
3-oxoacyl-(acyl-carrier-protein) reductase	124

**Table 3:** First 11 hub enzymes of the enzyme networks with corresponding reaction information

Gene	Enzyme Class	EC Number	Reaction
SOD2	Oxidoreductases	1.15.1.1	R00275: 2 O2.- + 2 H+ <=> Hydrogen peroxide + Oxygen
CYP2D14	Oxidoreductases	1.14.14.1	R02351: Estrone + Formate + Oxidized flavoprotein + H2O <=> 19-Oxoandrost-4-ene- 3,17-dione + Oxygen + Reduced flavoprotein R03697: Morphine + Oxidized flavoprotein + Formaldehyde + H2O <=> Codeine + Reduced flavoprotein + Oxygen R03087: Estradiol-17beta + Formate + Oxidized flavoprotein + H2O <=> 19- Oxotestosterone + Oxygen + Reduced flavoprotein
GGCX GC	Lyases	4.1.1.90	R05144: Gla protein + Vitamin K1 epoxide + H2O <=> Gla protein precursor + Phylloquinol + CO2 + Oxygen R09991: 2,3-Epoxymenaquinone + Gla protein + H2O <=> Menaquinol + Gla protein precursor + CO2 + Oxygen
ALDH7A1	Oxidoreductases	1.2.1.31	R04390: alpha-Aminoadipoyl-S-acyl enzyme + NADPH + H+ <=> L-2-Aminoadipate 6- semialdehyde + Holo-Lys2 + NADP+
MECR	Oxidoreductases	1.3.1.38	R06985: trans-Hex-2-enoyl-CoA + NADPH + H+ <=> Hexanoyl-CoA + NADP+ R07761: (2E)-Octadecenoyl-CoA + NADPH + H+ <=> Stearoyl-CoA + NADP+
QDPR	Oxidoreductases	1.5.1.34	R01794: Dihydrobiopterin + NADPH + H+ <=> Tetrahydrobiopterin + NADP+
FASN	Transferases	2.3.1.85	R05188: Acetyl-CoA + n Malonyl-CoA + 2n NADPH + 2n H+ <=> Long-chain fatty acid + n CO2 + 2n NADP+ + (n+1) CoA + n H2O
TXNRD2	Oxidoreductases	1.8.1.9	R09372: 2 NADPH + 2 H+ + Methylselenic acid <=> 2 NADP+ + 2 H2O + Methaneselenol
TXNRD1	Oxidoreductases	1.8.1.9	R09372: 2 NADPH + 2 H+ + Methylselenic acid <=> 2 NADP+ + 2 H2O + Methaneselenol
PTGR1	Oxidoreductases	1.3.1.-	R08754: Geranylgeranyl diphosphate + NADPH + H+ <=> Dihydrogeranylgeranyl diphosphate + NADP+ R08755: Dihydrogeranylgeranyl diphosphate + NADPH + H+ <=> Tetrahydrogeranylgeranyl diphosphate + NADP+ R08756: Tetrahydrogeranylgeranyl diphosphate + NADPH + H+ <=> Phytyl diphosphate + NADP+
FASN	Oxidoreductases	1.1.1.100	R10120: 3-Ketopimeloyl-(acp) methyl ester + NADPH + H+ <=> 3-Hydroxypimeloyl-(acp) methyl ester + NADP+ R10116: 3-Ketoglutaryl-(acp) methyl ester + NADPH + H+ <=> 3-Hydroxyglutaryl-(acp) methyl ester + NADP+ R07763: 3-Oxostearoyl-(acp) + NADPH + H+ <=> 3-Hydroxyoctadecanoyl-(acp) + NADP+

The activity of many enzymes can vary significantly according to the cells' metabolic activity and complex metabolic pathways [33-4]. As demonstrated in Table 3, the following genes CYP2D14 (1.14.14.1), MECR (1.3.1.38), FASN (2.3.1.85), and FASN (1.1.1.100) participate in fatty acid and lipid metabolism pathways. GGCX (4.1.1.90) and QDPR (1.5.1.34) participate in ubiquinone and other terpenoid-quinone biosyntheses as well as the metabolism of cofactors and vitamin pathways, whereas SOD2 (1.15.1.1) acts in FoxO signaling, transport and catabolism pathways. TXNRD2 (1.8.1.9), TXNRD1 (1.8.1.9), and ALDH7A1 (1.2.1.31) participate in the biosynthesis and degradation of amino acids (such as Glycine, Serine, Threonine, Lysine, Valine, Leucine, Isoleucine, Arginine, Proline, Histidine, Tryptoph, and Beta-Alanine), biosynthesis of fatty acids and lipid, glycerolipid metabolism, nucleotide and carbohydrate metabolism, and glycolysis/gluconeogenesis. Changes in these "gene expressions" could directly affect the production of the pathway. The abnormality among these enzymes that harms bovine health and reduces milk production may be attributed to carbohydrate and protein changes and lipid metabolism [11,12-32]. In the present work, enzymes were found to play critical roles in controlling or regulating cellular responses to specific physiological stimulus. This finding contributes to various experimental strategies used for the identification of protein interactions. Nevertheless, traditional methods are both costly and time consuming [2]. Thus, the reconstruction of a comprehensive enzymatic network for milk production in dairy cattle will increase our understanding of this complex metabolic process and enable animal geneticists and breeders to focus on key (hub) enzymes for ongoing breeding schemes. This can, in turn, result in a more rapid development in milk production studies and dairy industries [34-35].

It should be noted that although known metabolic data from different data sources were collected, many unknown factors could have effected the results of the present study. Further research is thus needed to identify and study these factors.

In the past decade, main advances have been made in the area of metabolic networks and their topological features. Such developments reveal new biological features and enable researchers to comprehend biological systems from a more abstract point of view. Moreover, studies involving the identification of metabolic networks are important as they help obtain a better understanding of mammary gland physiology as well as food, dairy, and animal sciences as related to mammary gland metabolic activity, milk composition, and milk quality. In the present study, we reconstructed and analyzed the enzyme centric network involved in milk production in cattle using information available through the KEGG metabolic pathway database. Characteristics of this network were analyzed, and the top 11 hub enzymes were identified. Cattle mammary gland scale-free behavior of the enzyme network suggests that during evolution, new nodes tend to have been attached preferentially to a few highly- connected ancient nodes. This possibly indicates that enzymes (nodes) with very high- degrees are likely to be very ancient, a finding which was further confirmed by the analysis of high degree nodes [38-37].

The results the present work include information that might improve our understanding of cow milk production and breeding. Analyses of enzyme networks are also used for the modeling and simulation of biological systems as well as designing desired phenotypes that can help increase the quality or quantity of dairy products.

## Notes

**Acknowledgments: **The authors thank Shiraz University for financial support and J. Freyhof for his comments and the *C. linea *photo.
